# Current applications of nanomaterials in urinary system tumors

**DOI:** 10.3389/fbioe.2023.1111977

**Published:** 2023-02-20

**Authors:** Zhounan Qian, Yang Zhang, Jie Yuan, Sun Gong, Binghai Chen

**Affiliations:** Department of Urology, Affiliated Hospital of Jiangsu University, Zhenjiang, China

**Keywords:** mesoporous silica nanoparticles (MSN), gold nanoparticles (Au NPs), carbon nanotubes, magnetic nanoparticles (MNP), urinary tumors, quantum dots, liposome

## Abstract

The development of nanotechnology and nanomaterials has provided insights into the treatment of urinary system tumors. Nanoparticles can be used as sensitizers or carriers to transport drugs. Some nanoparticles have intrinsic therapeutic effects on tumor cells. Poor patient prognosis and highly drug-resistant malignant urinary tumors are worrisome to clinicians. The application of nanomaterials and the associated technology against urinary system tumors offers the possibility of improving treatment. At present, many achievements have been made in the application of nanomaterials against urinary system tumors. This review summarizes the latest research on nanomaterials in the diagnosis and treatment of urinary system tumors and provides novel ideas for future research on nanotechnologies in this field.

## 1 Introduction

The development of nanotechnology has brought new breakthroughs in the diagnosis and treatment of cancer (Wakaskar, 2018; Chaturvedi et al., 2019; Khan et al., 2020). Nanoparticles are usually loaded with therapeutic drugs, proteins, photothermal agents, imaging agents or immune molecules (Liu et al., 2018). Nanoparticles are modified on demand, and those with recognizable ligands are easily taken up by target cells after receptor‒ligand interactions (Song et al., 2017). Nanoparticles are powerful drug carriers and can increase drug uptake by tumors by prolonging drug circulation and reducing the risk of adverse toxic effects on nearby healthy tissue (Ahmed et al., 2012).

Urinary system tumors are common malignant tumors in humans with gradually increasing incidence (Siegel et al., 2019). The treatment of urinary system tumors is diverse. For example, chemotherapy and radiotherapy are the main treatment strategies for advanced renal cancer. However, drug resistance is very common (Penticuff and Kyprianou, 2015). Nanotechnology can be used to enhance the therapeutic effects of cancer drugs (Jaishree and Gupta, 2012). Although targeted therapy and immunotherapy are widely used, resistance to these modalities is inevitable, and the development of nanomaterials provides an alternative direction. Moreover, traditional treatment methods, such as surgery, radiotherapy and endocrine therapy, are facing great challenges. The development of nanomaterials provides novel ideas for the treatment of urinary tract tumors. Thus, this review summarizes the applications of several nanomaterials commonly used to treat urinary tract tumors.

## 2 Mesoporous silica nanoparticles (MSNs)

MSNs have well-defined mesoporous structures with diameters ranging from 2 to 10 nm, large pore volumes of 0.6–1 cm^3^/g and high surface areas of 700–1,000 m^2^/g (Iturrioz-Rodriguez et al., 2019). MSNs have certain characteristics, such as a uniform structure, large surface area, and modifiable pore size, and work as a suitable repository for loading therapeutic/diagnostic agents. MSNs protect their cargo from premature release and subsequent undesirable degradation in the stomach and intestine until they reach the target location (He and Shi, 2014; Nawaz et al., 2018). MSNs in the size range of 50–300 nm can promote endocytosis in living animal and plant cells without producing any significant cytotoxicity. In drug delivery and cancer treatment, the particle size of nanocarriers must be less than 100 nm to circulate in blood vessels for a long time and serve as a passive drug delivery carriers based on enhanced permeability and retention (EPR) effects. On the other hand, the pore sizes of MSNs can be adjusted from 2 to 6 nm, which allows the loading of different drug molecules. Therefore, MSNs have high drug loading and packaging efficiency (Porrang et al., 2022).

### 2.1 Application of MSNs in PCa

**FIGURE 1:**
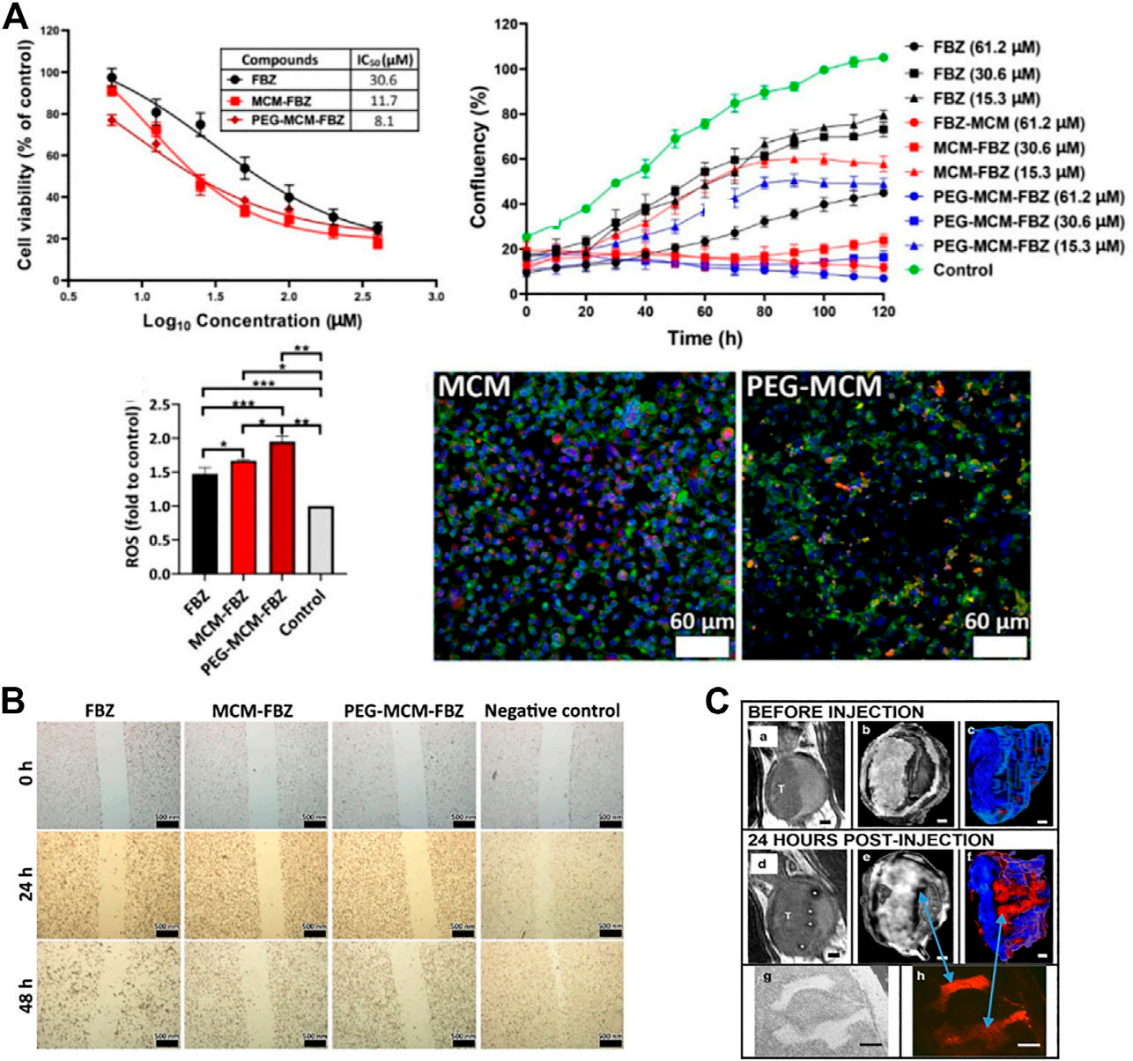
Synthesis and applications of mesoporous silica nanoparticles (MSNs). **(A)** The cytotoxicity of FBZ increased when it was bound the nanomaterial. Moreover, the inhibition of prostate cancer (PCa) cell proliferation was significantly increased. The intracellular ROS contents were also increased, thus inhibiting the proliferation of tumor cells. Fluorescence experiments showed that the FBZ complexes, especially the PEG-MCM-FBZ complex, increased the uptake of FBZ. **(B)** The complex effectively inhibits the migration of PCa cells. **(C)** Bladder tumor cells absorbed PEG-Gd_2_O_3_-TRITC-MSN particles with higher affinity than normal bladder epithelial cells and can be used for MRI T2 scanning.

MSNs can be used in the treatment of PCa. Many researchers have used MSNs as carriers to load therapeutic drugs, and MSNs can deliver these drugs to tumor cells. For example, the study of Zanib Chaudhary et al. investigated resveratrol (RES), a polyphenol with antitumor properties. However, its poor pharmacokinetics and stability and low solubility limit its clinical application. Loading RES onto MSNs can significantly improve the efficacy of RES in PCa (Chaudhary et al., 2019). Fenbendazole (FBZ) is a potential anticancer drug whose application is limited by its low water solubility. The water solubility of FBZ can be increased when serialized on β-lactoglobulin-modified MSNs, which can increase the cytotoxicity of FBZ and inhibit the migration of PCa cells (Koohi et al., 2022). (Figures 1A,B) It is also possible to serialize FBZ on pegylated MSN (MCM-41) to improve its cytotoxicity and increase its delivery to PCa cells (Esfahani et al., 2021).

Studies have found that functionalized MSNs themselves can also inhibit tumors. *Walterinnesia aegyptia* venom (WEV) serialized on MSNs is more effective than WEV alone. Researchers have also demonstrated that snake venom silica nanoparticles could alter the cell cycle in PCa cells and accelerate cell apoptosis. A study showed that the continuous delivery of nanoparticles carrying WEV is an effective treatment for PCa (Badr et al., 2013). pH-sensitive delivery systems can provide on-demand and selective drug release at acidic tumor sites. Polyacrylic acid (PAA)-functionalized MSNs (PAA-MSNs) can be used as an effective pH response template with great potential for effective controlled release in cancer therapy. PAA-MSNs also showed strong cellular uptake and a longer circulation time *in vivo*, and good results were obtained when they were linked to bicalutamide for the treatment of PCa (Saroj and Rajput, 2019). Researchers also linked etoposide (ETS) to PAA-MSNs, which greatly increased the cytotoxicity of ETS (Saroj and Rajput, 2018). Moreover, when calcium peroxide (CaO_2_) was loaded into PAA-MSNs, free CaO_2_ without antitumor effects can release reactive oxygen species (ROS) in response to the acidic microenvironment. This induces mitochondria-mediated apoptosis through significant oxidative stress, minimizes damage to normal tissues, and exerts excellent antitumor effects. This system represents a new way to treat PCa (Wu et al., 2020).

MSNs can also be used to diagnose PCa. Researchers developed an electrochemical immunosensor based on MSNs (Wang et al., 2013). Other researchers designed MSNs containing pH indicator molecules for PCa detection, which can improve the sensitivity of detecting prostate-specific antigen (PSA) (Shao et al., 2018). PSA-targeted manganese-oxide MSNs were found to be useful for detecting PCa, as they can accumulate in PCa cells but not in non-cancerous cells. PCa can be visualized using PSA-targeted fluorescence and Magnetic Resonance (MR) dual-function nanoparticles. They can be used as an MR contrast agent (Du et al., 2020). It is generally harder to detect low molecular weight biomarkers *in vivo* by traditional spectrometric methods, as they often exist in low concentrations and are covered by many other proteins. In fact, studies have shown that less than 25% of PCa cases with increased PSA levels were diagnosed appropriately.

In addition, a second biopsy can diagnose PCa in approximately 30% of patients with a previously benign biopsy result. Researchers have designed diagnostic systems for PCa. These use mercaptan MSN (MSN-SH) to detect low molecular weight proteins and distinguish between patients with benign prostatic hyperplasia and those with PCa with elevated PSA levels (Vidaurre-Agut et al., 2019).

### 2.2 Application of MSNs in bladder cancer

Researchers have linked targeted doxorubicin (DOX) to MSNs and the peptide conjugate CSNRDARRC (DOX-loaded MSNs@PDA-PEP) to form a complex. Further study suggested that the complex significantly improved cell uptake efficiency compared with DOX alone (Wei et al., 2017). Researchers invented a compound based on MSNs and found that 70% of bladder cancer cells were labeled by nanoparticles. The tumor contrast was enhanced by the nanoparticles, which could be used for tumor staging, treatment monitoring and drug delivery (Sweeney et al., 2016). At present, there are many studies on microRNAs, indicating that these RNAs have antitumor effects. Researchers have reported combinations of microRNA and MSNs as delivery agents. The uptake of these multifunctional nanoparticles by bladder cancer cells overexpressing EGFR was enhanced *via* receptor-mediated cellular internalization (Haddick et al., 2020). Moreover, modified MSNs were constructed and used as delivery carriers to deliver both miRNA-34a and si-PD-L1 to bladder cancer cells. Notably, the nanoparticles were biocompatible, which protects the compound from degradation. Therefore, this system could be used as a treatment for bladder cancers (Shahidi et al., 2022).

### 2.3 Application of MSNs in renal cancer

Lonidamine (LND), a heat-sensitive inhibitor of mitochondrial metabolism, was used in combination with photothermal-polydopamine (PDA) in the treatment of renal cell carcinoma (RCC). LND and PDA were loaded into MSNs with a star shape. The results indicated that the complex had excellent tumor targeting ability. LND and PDA combined with laser treatment enhanced the antiproliferation and anticancer abilities with good biocompatibility (Chen et al., 2021).

## 3 Gold nanoparticles (AuNPs)

Gold nanoparticles (AuNPs) have attracted great attention in biomedicine (Pissuwan et al., 2011; Dykman and Khlebtsov, 2012). AuNPs have large surfaces and different properties and are of great potential in diagnosis, delivery, photothermal and radiation therapy, enzyme fixation, and cell imaging (Zhang et al., 2010; Li et al., 2011; Nimesh et al., 2011; Pissuwan et al., 2011). AuNPs come in very diverse shapes, although spherical nanoparticles are thought to be the main type. Depending on the production method, AuNPs can be made in different forms: triangles, hexagons, octahedrons, cells, nanospheres, pores, stars, and nanorods (Ovais et al., 2017). The different form determines what the AuNP carries (Boisselier and Astruc, 2009). AuNPs can interact with biological fluids, and the nanoparticles acquire a biological component known as a protein corona (PC) (Maiorano et al., 2010; Corbo et al., 2016; Rampado et al., 2020). Thus, AuNPs can be widely used in the medical field.

### 3.1 Application of AuNPs in PCa

**FIGURE 2:**
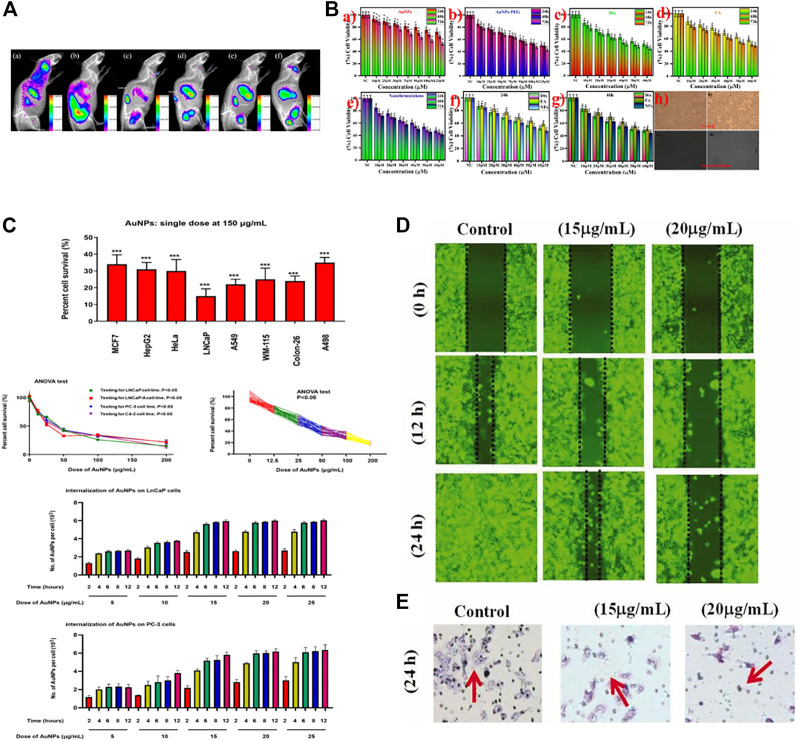
Applications of gold nanoparticles (AuNPs) in cancer. **(A)** PCA cells were treated with ultrafine AuNPs containing the gastrin-releasing peptide receptor. After 72 h, up to 6% of the AuNPs remained in the body, which could be used to detect PCa. **(B)** Docetaxel-coated AuNPs showed toxicity to PCa cells *in vitro*. **(C)** AuNPs were found to be toxic to eight types of tumor cells, most notably to PCa cells, showing toxic effects to all PCa cells tested. **(D)** AuNPs synthesized from abies fir plant extracts inhibited bladder cancer T24 cell migration in a concentration-dependent manner. **(E)** AuNPs mediated T24 cell death.

AuNPs can be used to detect PCa. Researchers designed a sensitive electrochemical immunosensor for the determination of PSA. AuNP/aminothiol-functionalized graphene-oxide composites were constructed and modified before they were used as an immunosensor platform to elevate the amount of PSA antibody 1 (Ab1) for PSA determination (Medetalibeyoglu et al., 2020). Studies have detected PCA3, a potential urinary biomarker, in urine using AuNPs (Htoo et al., 2019). Based on supramolecular hydrogel-AuNP spheres, an imaging biosensor with high sensitivity and specificity was developed for the determination of exosomes derived from PCa cells (Chen et al., 2020). The gold nanoprobes consisted of a specific peptide, and the AuNPs were used for the determination of PSA (Li et al., 2019a). Studies evaluated AuNPs *in vitro* and *in vivo* and found that these nanoparticles showed good potential features for cancer cell uptake and biodistribution and could also be used to detect PCa by fluorescence imaging (Pretze et al., 2018). (Figure 2A)

The application of AuNPs in the treatment of PCa is also very extensive. AuNPs can be used as carriers to deliver antitumor drugs to PCa cells. Researchers synthesized novel AuNPs conjugated with abiraterone (AuNPs-AB), which have shown good prospects for the treatment of PCa (Stolarczyk et al., 2020). Docetaxel (Dtx) was encapsulated by AuNPs for delivery to PCa cells (Thambiraj et al., 2021). A synthetic Gen-AuNP conjugate (Gen@AuNPs) selectively inhibited the proliferation of PCa cells. The authors also demonstrated the stability and bioactivity of the AuNPs along with their low toxicity to normal cells (Vodnik et al., 2021). Phosphatidylserine (PS) is an essential lipid that mediates macrophage exocytosis and is dysregulated in tumors. Biomimetic phosphatidylserine-coated AuNPs (PS-AuNPs) were synthesized, and their potential in PCa was investigated *in vitro*. After evaluating histone-associated DNA fragments as a marker of apoptosis, the researchers found that DNA fragmentation was significantly increased after PS-AuNP treatment compared with control treatment. Therefore, PS-AuNPs were indicated as a potential PCa treatment (Radaic et al., 2021).

AuNPs themselves can also be therapeutic for PCa. AuNPs have been shown to exert powerful cytotoxic effects, with synthetic AuNPs showing the most significant efficacy against PCa, whether or not the tumor cells were androgen-dependent. Further studies on the molecular mechanism showed that AuNPs can trigger the secretion of anticancer factors and myeloid cell-polarizing factors from tumor cells through MMP9 inhibition, thereby achieving antitumor effects (Hao et al., 2021). (Figure 2C) The same AuNPs decay at a high energy level and their radiation can be delivered to the tumor site without destroying the normal tissues or organs. Using AuNPs is a promising way to treat PCa (Al-Yasiri et al., 2019). AuNPs have also been widely used to treat PCa due to their unique optical properties. A silicon dioxide-coated AuNP cluster was proposed to address the therapeutic limitations of individual AuNPs and utilize their photothermal effects to treat typical PCa PC-3 cells. AuNP clusters have shown excellent therapeutic effects in photothermal tests under near-infrared radiation (Kim et al., 2020).

The combination of AuNPs and Gd(III) provides better cancer inhibition after radiotherapy. Precise tumor targeting by PSMA-1-modified AuNPs can achieve precise radiotherapy, reduce the irradiation dose, and minimize side effects (Luo et al., 2020). AuNPs are also effective radiosensitizers, and RALA (a short amphiphilic peptide)/AuNPs were synthesized, providing functional evidence of RALA-AuNP nuclear accumulation. RALA-AuNP produced meaningful radiosensitization using low microgram AuNP treatment concentrations (Bennie et al., 2021).

### 3.2 Application of AuNPs in RCC

Few studies of AuNPs in RCC have been reported. Researchers have found that 200 nm AuNPs have an antitumor effect in 786-O RCC cells by promoting cell apoptosis and inhibiting proliferation (Zhao et al., 2020). C. wenyujin is the main component of turmeric and has antioxidant, antiproliferative and antitumor properties. Researchers used C. wenyujin to synthesize AuNPs and found that the produced CW-AuNPs could activate the proapoptotic proteins caspase-3, caspase-9, Bid and Bad in renal cancer A498 cells *in vitro*. CW-AuNPs can reduce the level of Bcl-2 and thereby induce apoptosis of A498 cells (Liu et al., 2019). Other studies have found that AuNP treatment reduces the viability of cancer cells, suggesting a potential alternative to nephrectomy (Nikzad et al., 2017).

### 3.3 Application of AuNPs in bladder cancer

Hyaluronidase (HAase) is a bladder cancer biomarker found in urine. HAase can prevent the redshift observed when cationic gold nanoparticles (CTAB) aggregate with other substances. Therefore, this color change can be used for the diagnosis of bladder cancer (Nossier et al., 2014). Other researchers considered urine hepatocellular carcinoma upregulated RNA (HURP) as a biomarker of bladder cancer and developed a AuNP assay to directly detect unamplified HURP RNA for bladder cancer diagnosis (Eissa et al., 2014).

AuNPs can also be used in the treatment of bladder cancer. Researchers found that by combining fir extract with AuNPs, apoptosis of tumor cells could be promoted to play an anticancer role (Wu et al., 2019). (Figures 2D,E) Because AuNPs are effective enhancers of radiotherapy, some researchers improved the control of locally advanced bladder cancer. They used AuNPs to locate bladder tumors and found that the AuNPs were distributed throughout the bladder wall. However, most AuNPs are related to extracellular keratins, and their localization in the tumor stroma contributes to their specific radiotherapy enhancement for muscle-invasive bladder cancer (Smilowitz et al., 2017). AuNPs themselves also have therapeutic effects. Researchers have studied the cancer suppression effects of AuNPs in 5637 bladder cancer cells exposed to different concentrations of AuNPs for 24 h. The results showed that the AuNPs could reduce the survival of 5637 cells in a dose-dependent manner. In addition, ROS production was significantly increased in cells treated with 25 and 50 μg/mL AuNPs. These AuNPs could promote the apoptosis of tumor cells by inducing Bax overexpression and downregulating Bcl-2 (Daei et al., 2022).

## 4 Quantum dots (QDs)

**FIGURE 3:**
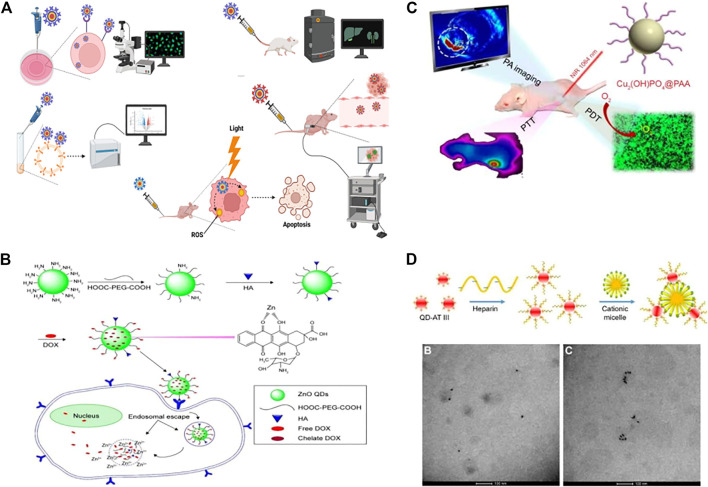
Biomedical applications of quantum dots (QDs). **(A)** QDs were used for intracellular imaging, *in vivo* imaging, fluorescence-activated cell sorting, photodynamic therapy, and traceable drug delivery. **(B)** QDs were used for drug delivery, such as the hyaluronic acid-ZnO QD-polyethylene glycol (HA-ZnO-PEG) material. This drug delivery system can release DOX (doxorubicin) in acidic cells. **(C)** QDs can be used for imaging and enable a combination of photodynamic therapy and photothermal therapy as well as photoacoustic imaging. **(D)** QDs have been used in sensors. TEM images of QDs-AT III-heparin without CTAB can be easily distinguished from TEM images of QD aggregates with CTAB after quantum dot synthesis and polymerization.

Quantum dots (QDs) are nanomaterials with diameters of 2–10 nm (Sukhanova et al., 2018). QDs have unique physical and chemical properties, including high stability. Moreover, most QDs are considered non-toxic, so they are widely used in various fields of medicine (Rajendiran et al., 2019). QDs are potential smart drug delivery tools that can be used in cancer therapy, and their photostability, tunable emission, and wide excitation range make them more effective fluorescent markers than organic dyes in biological applications (Breus et al., 2015; Manshian et al., 2017; Hossen et al., 2019). (Figure 3A)

### 4.1 Application of QDs in PCa

Researchers synthesized highly stable AgInSe/ZnS QDs that can selectively target PCa and be taken up in large amounts. Therefore, AgInSe/ZnS QDs can be used as imaging probes to guide diagnosis and treatment (Ncapayi et al., 2021). (Figures 3B–D) Researchers used yeast cells and *Saccharomyces cerevisiae* to synthesize cadmium telluride (CdTe) QDs in modified Czapek medium through biological methods. *In vitro* experiments showed that the CdTe QDs induced a significant increase in ROS levels in PC-3 cells. Moreover, CdTe also arrested PC-3 cell growth in the G2/M phase of the cell cycle. Thus, CdTe QDs induced cell death and nuclear apoptosis in a dose-dependent manner (Jigyasu et al., 2020). Nanocomplexes containing QDs and β-cyclodextrin have been synthesized. This nanoconjugate has folate-targeting properties and has been used to deliver the anticancer Compound C-2028 to PCa cells. The QDs enter the cell through multiple endocytic pathways with various efficiencies to deliver the drug to cancer cells (Pilch et al., 2022). Researchers designed and synthesized a nanoparticle probe PSMA receptor-targeted QD (PSMA-QD655) by combining functionalized amino-PEG QDs with DUPA-targeted polypeptide constructs through heterobifunctional joints. PSMA-QD655 is a near-infrared imaging agent that can be used for navigation during surgery (Asha et al., 2021). Through the electrostatic adsorption of a large number of QDs on superparamagnetic Fe_3_O_4_, researchers prepared red and green magnetic QD nanobeads (MQBs) with excellent magnetic and high luminescence properties. MQBs can be used as multifunctional probes for the simultaneous determination of free and total PSA (Rong et al., 2019).

### 4.2 Application of QDs in bladder cancer

QDs have also been widely used in bladder cancer. Researchers prepared QD fluorescent probes conjured with prostate stem cell antigen (PSCA) monoclonal antibody (QD-PSCA). The probe specifically identifies PSCA expressed in bladder cancer cells. The stable fluorescence of the probe can be used as a specific marker (Yuan et al., 2018). A nanocarrier system was constructed with Mn:ZnS QDs. This delivery system can improve drug delivery efficacy in bladder cancer (Manan et al., 2021). The molecular detection of malignant nuclear matrix protein 22 (NMP22) in the urine has been introduced into clinical practice to identify bladder tumors. Researchers have also designed two-color QDs that can detect NMP22 with high sensitivity (Othman et al., 2020).

### 4.3 Application of QDs in RCC

Black phosphorus QDs (BP-QDs) can enhance the apoptosis of RCC cells after ionizing radiation (IR), indicating that BP-QDs have potential application value in the RCC radiosensitization therapy (Lang et al., 2022). Researchers have measured the expression of the proteins Tiam1 and Rac1 in RCC using immunohistochemistry (IHC) and QD labeling methods. It was found that the expression levels of Tiam1 and Rac1 are related to the differentiation, staging and lymphatic metastasis of RCC, suggesting that they are critical in RCC invasion and metastasis (Shan et al., 2017).

## 5 Carbon nanotubes (CNTs)

**FIGURE 4:**
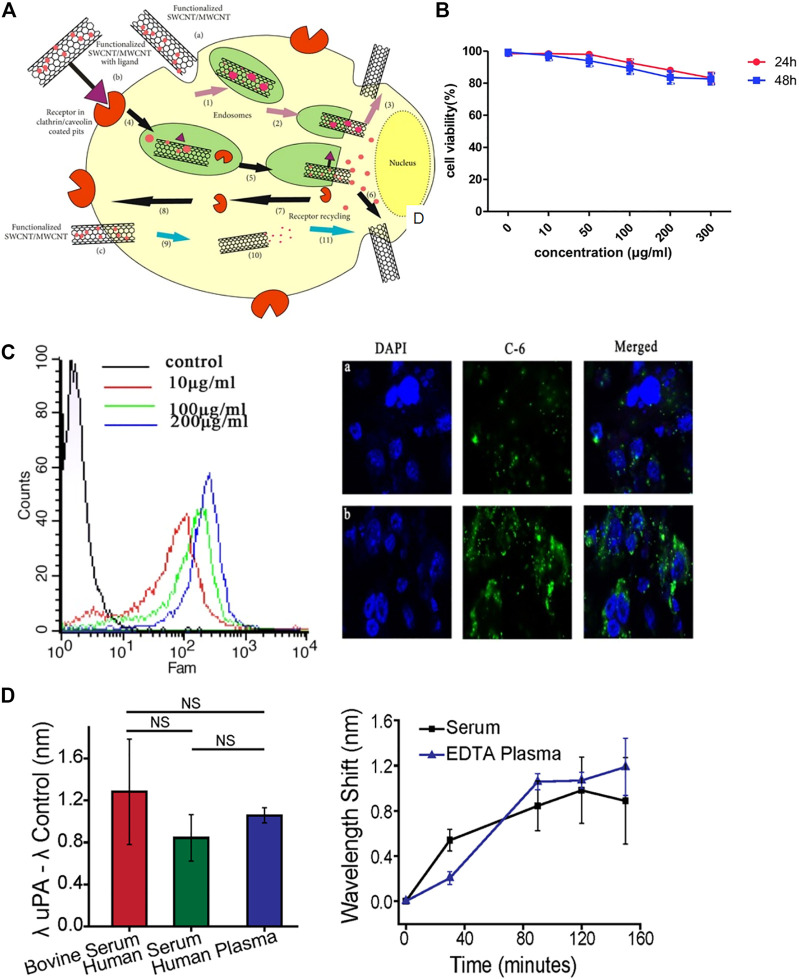
Biological applications of carbon nanotubes (CNTs). **(A)** CNTs penetrate into cells through various ways, including non-receptor-mediated endocytosis, receptor-mediated endocytosis and endocytosis-independent methods. **(B)** Flow cytometry and immunofluorescence assays indicated the uptake of CNT-PEG. **(C)** Cytotoxicity of CNT-PEG uPA at different concentrations. **(D)** Sensor detection of uPA in human blood products.

Carbon nanotubes (CNTs) are sheets of graphene 1 nm in diameter and a few microns in length. Single-walled CNTs (SWCNTs) and multiwalled CNTs (MWCNTs) (Elhissi et al., 2012) are the two common types of CNTs. Functionalized CNTs have the ability to cross the cell membrane, which allows functionalized CNTs to target specific tumor cells through endocytosis (Lacerda et al., 2012). (Figure 4A) Modified CNTs can be used to evaluate treatment, cell survival and apoptosis (Rastogi et al., 2014).

### 5.1 Application of CNTs in PCa

A nanoparticle delivery system including MWCNTs conjugated with the polypeptide H3R6 has been discovered for PCa immunotherapy in which the immunotherapeutic agents can be efficiently delivered to the prostate and lymph nodes (Xia et al., 2018). A novel nanoultrasound contrast agent based on MWCNTs was reported. Compared with traditional contrast materials, this contrast agent provides better visibility and accuracy and can target PCa cells more effectively (Gu et al., 2018). (Figures 4B,C) Researchers designed an optical sensor based on the fluorescence characteristics of SWCNTs. These sensors are expected to be used to distinguish between invasive PCa and inert prostate cells and reduce excessive treatment options for this disease (Williams et al., 2018). (Figures 4D,E)

### 5.2 Application of CNTs in bladder cancer

Bladder tumor-specific SWCNTs are delivered to the bladder at a low dose. Then, the nanotubes are heated with 360° NIR light for 30 s. NIR light can heat the tumor while protecting the healthy bladder wall (Virani et al., 2018). Epirubicin (EPI) was loaded on magnetic MWCNTs (MMWCNTs-EPI) for intravesical infusion. The MMWCNT-EPI system has high efficiency in enhancing cytotoxicity and inhibiting proliferation *in vitro* and *in vivo*, showing potential clinical application value (Suo et al., 2019).

## 6 Liposomes

**FIGURE 5:**
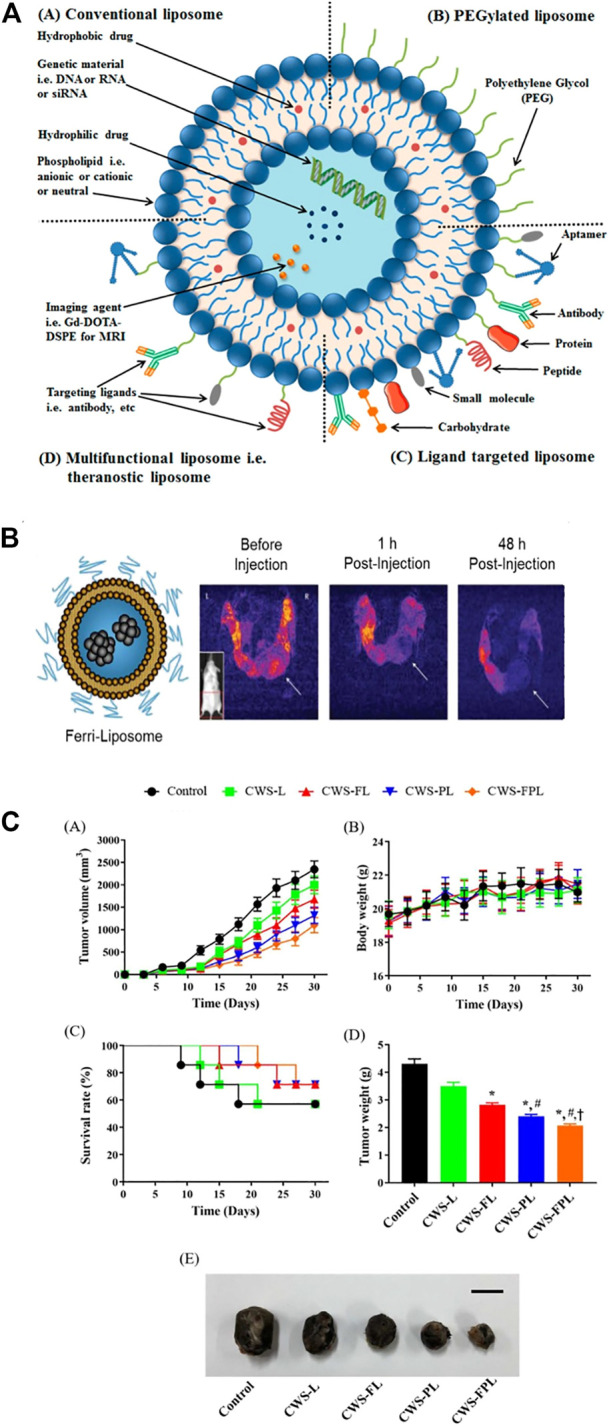
Liposomes have been used for cancer diagnosis and therapy. **(A)** A specific targeting ligand was incorporated to modify the surface of PEGylated liposomes. **(B)** T2-weighted MR images of a transgenic mouse before and after injection of Ferri-liposomes. **(C)** An *in vivo* study indicated that various liposomal formulations inhibited tumor growth.

Liposomes are spherical structures with sizes ranging from 25 nm to 2.5 μm (Akbarzadeh et al., 2013) Liposomes can be structured as monolayer (ULV) or multilayer (MLV). ULVs are suitable for the encapsulation of hydrophilic drugs or antigens. MLVs feature multiple lipid bilayers, which act as barriers to resist enzymes, certain pH conditions, and free radicals *in vivo*. Thus, liposomes prevent drug degradation until its release at target cells, organs, or systems. Due to their high biocompatibility, degradability, low toxicity, and ability to encapsulate hydrophilic and hydrophobic compounds, liposomes represent the most successful drug delivery system because of their high biocompatibility, degradability and low toxicity (Felice et al., 2014; Bozzuto and Molinari, 2015). (Figures 5A,B)

### 6.1 Application of liposomes in PCa

The considerable side effects of the anthracycline doxorubicin (DOX) are major drawbacks. Simvastatin (Sim) can be used in combination with DOX. Researchers combined DOX and Sim on herceptin-conjugated liposomes. Studies have shown that liposomes can target PCa cells and exert an antitumor effect (Li et al., 2019b). Antisense oligonucleotides (ASOs) have a significant advantage due to their ability to provide virtually unlimited targeting of any gene. However, ASOs have the disadvantage of poor delivery *in vivo*. Researchers used the penetrating peptide iRGD to functionalize liposomes as anti-androgen receptor (AR) vectors. IRGD/liposomes significantly elevated the concentration of AR/ASO in target tissues and therefore inhibited tumor growth (Guan et al., 2021).

Liposomes increase the solubility the complexes, enhance the cytotoxicity of PCa drugs, and significantly reduce the growth rate of PCa cells, resulting in high radiotherapy efficacy in patients with locally advanced PCa (Silva et al., 2021). PSMA is a marker used for the diagnosis of advanced PCa and is recognized for targeted drug delivery. A liposome composed of a PSMA ligand and polyethylene glycol was synthesized. Liposomes with small PSMA binding motifs can be used to specifically identify PSMA + PCa cells (Yari et al., 2019).

### 6.2 Application of liposomes in bladder cancer

The BCG cell wall skeleton (BCG/CWS) was encapsulated in liposomes. Specific ligands were then conjugated on the surface of the liposomes to facilitate targeting and cell penetration. The modified liposomes significantly elevated the endocytosis of BCG/CWS by bladder cancer cells and strengthened the antitumor effect. Thus, BCG/CWS-modified liposomes could be a potential, highly efficacious therapeutic candidate in bladder cancer. (Figures 5C,D). The accumulation and distribution of DOX within liposomes in the bladder are significantly higher than those of intravenously administered DOX (Mikhail et al., 2017). Researchers developed a cationic liposome that combines three subclasses of mycolic acid (MA). This liposome can be effectively absorbed by MB49 bladder cancer cells and then induce antitumor immunity *in vivo* (Yoshino et al., 2019).

### 6.3 Application of liposomes in RCC

A liposome specifically targeting tumors was synthesized with phospholipids and cholesterol and showed excellent tumor-specific uptake as well as tumor inhibition (Pal et al., 2019).

## 7 Magnetic nanoparticles (MNPs)

Magnetic nanoparticles are an important class of nanoparticles that are usually made of pure metals (Fe, Co., and Ni) or mixtures of metals and polymers. The utilization of MNPs has increased in hyperthermic cancer treatment, controlled drug release, magnetic resonance imaging, and biosensing. A key advantage of MNPs is their ability to be magnetically manipulated by external magnetic fields (Farzin et al., 2020). MNPs can alter drug pharmacokinetics to reduce cytotoxicity and increase the drug release rate. In addition to the possibility of localizing MNPs to cancer cells by the application of magnetic fields, MNPs can be modified with high-affinity ligands. Additionally, MNPs can be applied to improve the MRI image contrast in the target tissue. MNPs can be localized to tissue sites to increase proton relaxation and improve visibility (Sun et al., 2008).

### 7.1 Application of MNPs in PCa

**FIGURE 6:**
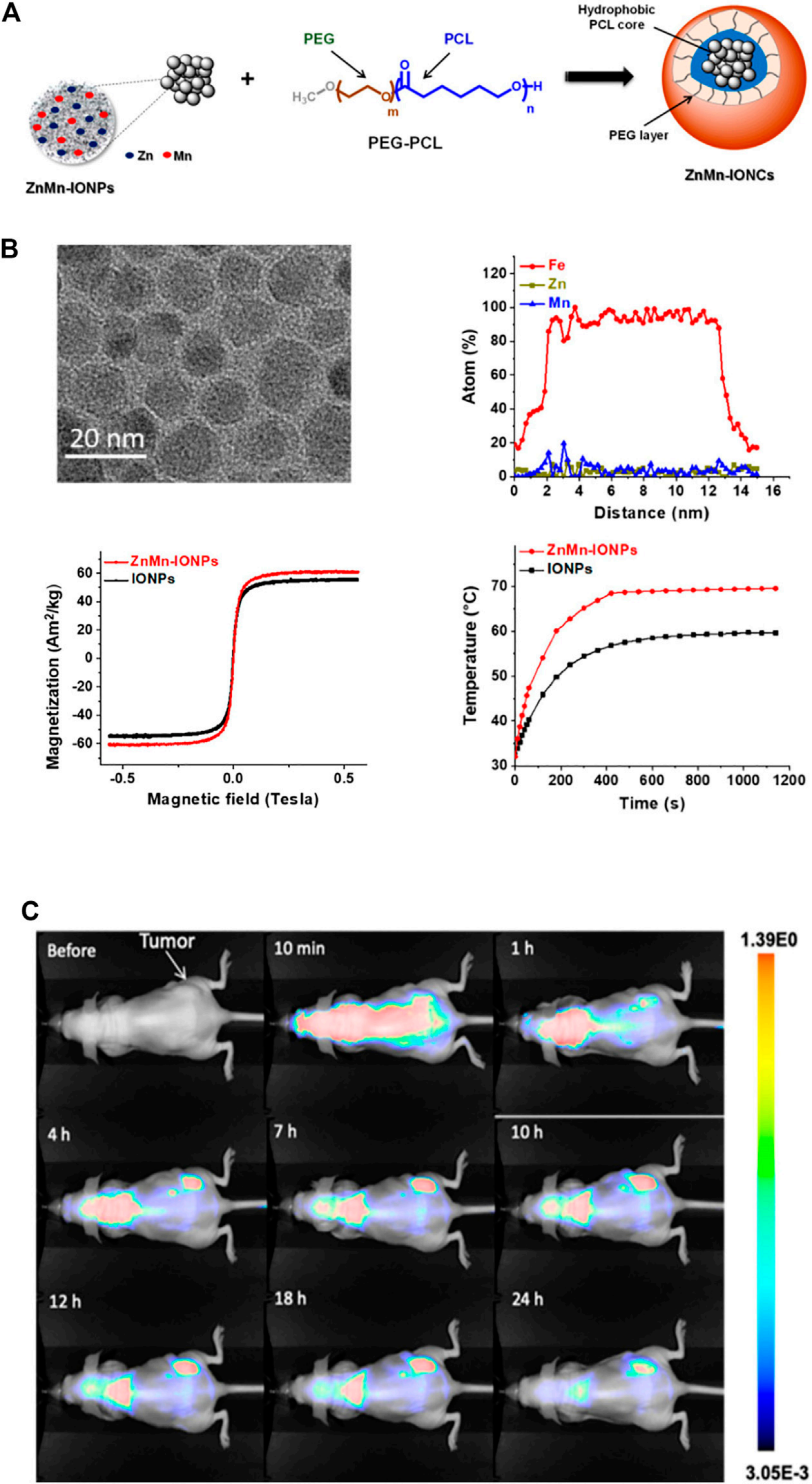
Biological applications of magnetic nanoparticles (MNPs). **(A)** Iron oxide nanoparticles with Zn and Mn were encapsulated into a nanocarrier. **(B)** Enhanced heating efficiency was achieved upon exposure to an alternating magnetic field. **(C)** *In vivo* evaluation of the nanoparticles suggested that ZnMn-IONCs started to accumulate in tumors. The fluorescence intensity in the tumors was higher than that the liver, kidneys, spleen and lungs.

Magnetic hyperthermia (MHT) is a promising treatment for solid tumors. MHT generates heat in the presence of an alternating magnetic field (AMF). Researchers found new magnetic nanoparticles with enhanced heating efficiency that generate ideal intratumoral temperatures when exposed to an AMF. *In vivo* experiments confirmed that the nanoclusters significantly accumulate in tumor sites within hours and elevate the temperature to more than 42°C after exposure to an AMF. Finally, MHT application showed great inhibition of PCa cell growth with no side effects (Albarqi et al., 2020) (Figure 6). Galbanic acid, a natural sesquiterpenoid coumarin compound, causes significant toxicity to LNCaP PCa cells. Galbanic acid was loaded onto Fe_3_O_4_-coated MNPs and showed significant toxicity to all PCa cells (Mohtashami et al., 2019) Researchers also developed multifunctional MSNs that bind photosensitizers. These MSNs demonstrated significant anticancer effects by inducing apoptosis in PCa cells (Choi et al., 2018).

## 8 Discussion

The development of nanotechnology and nanomaterials for the diagnosis and treatment of urinary system tumors has brought great achievements and opportunities (Table 1). Many of these studies are still in the experimental stage, and many of these concepts have not reached clinical application. There have been a few reports on nanotechnology and nanomaterials in bladder cancer and kidney cancer; however, we are far from fully understanding these technologies. We believe that through the efforts of researchers and clinicians, more diagnosis and treatment methods will be invented for patients with urinary tract tumors.

Despite the therapeutic benefits, problems of nanotechnology and nanomaterials in terms of biological safety as well as metabolism *in vivo* should be given sufficient attention. Nanomaterials vary in their distribution, which depends on their shape, size, porosity, surface functionalization, etc. (He et al., 2011). For instance, MSNs are mainly present in the liver and spleen (Fu et al., 2013). AuNPs are primarily concentrated in the liver, spleen and bone marrow (Blanco et al., 2015). CNTs are different from other nanomaterials. They enter the cells in two ways, through endocytosis and passive diffusion, while QDs are mainly distributed and accumulate in the reticuloendothelial system and kidney. These differences in distribution result in various metabolic fates, applications and toxicities. As far as metabolism is concerned, first, the clearance of nanomaterials, including MSNs and AuNPs, depends on clearance by the kidney and biliary tract. Second, nanomaterial metabolism depends on their size. Small AuNPs (less than 10 nm) are easier to metabolize than large AuNPs (Zhou et al., 2011). QDs with larger particle sizes remain unchanged *in vivo* for a longer time and are more difficult to remove. In addition, nanomaterials can combine with specific proteins and be found in systemic circulation. Thus, these nanomaterials can be absorbed by phagocytic cells in the liver and spleen, such as liver Kupffer cells and splenic B Cells. Finally, the nanomaterials can be excreted from the body after phagocytosis by the macrophages in the alveoli (Huang et al., 2011). In addition to their dosage, the particle size of nanomaterials has a great impact on their toxicity (Greish et al., 2012; Shao et al., 2017). Moreover, the nanoparticle surface properties are the most important factor that affects toxicity. Nanoparticles with positive charges are more likely to induce an immune response and cytotoxicity than neutral or negatively charged nanoparticles (Pisani et al., 2017; Li et al., 2019c). The cytotoxicity of nanoparticles is also considered to be shape-dependent (Lopez-Chaves et al., 2018) and depends on the biomolecules present on their surface (Xie et al., 2017).

Many problems with nanoparticles remain to be solved before these new developments can be applied in clinical settings. First, how can we improve the targeting efficiency of nanoparticles? Higher efficiency indicates that lower doses can be used with less toxicity. Second, we need to try to reduce particle loss in the targeting process, which is another way to increase efficiency and reduce toxicity. Third, and one of the most important issues, is how to reduce the side effects caused by nanoparticles, which usually lead to impairment of the kidney and liver. Fourth, we should also pay increasing attention to simplifying the manufacturing of nanoparticles so that their cost could be lower for wide use in the clinic. Finally, the waste from the production of these nanoparticles may pollute the environment, and we should thus optimize the production process to reduce this threat.

Furthermore, more clinical studies need to be conducted based on the issues mentioned above. For example, it is still unknown whether patient characteristics (e.g., age, sex, tumor type, tumor location, previous treatment) will alter the effects of some nanoparticles. In addition, it is also unknown whether nanoparticles can induce acute exacerbation of other diseases, such as asthma and diabetes. Third, nanoparticle application requires a complete set of platforms, which report the intake, distribution and metabolism of the drug in the body. Finally, most of the current research is focused on the design, production and targeting of nanoparticles to treat tumors. We believe that patients could benefit more from novel clinical research, including nanoparticle localization, which is also crucial in tumor therapy, for continuous anticancer effects.

At present, we are only beginning to study nanoparticles, most of which have not been put into clinical application. More clinical studies will have to be done to assure safety and effectiveness. Although many studies have shown the advantages of nanoparticles in the diagnosis and treatment of tumors, their potential biosafety and toxicity should also be given more attention. More critical questions should also be answered, including whether the age, weight, basic disease status, etc. of patients influence the effects of nanoparticles. Therefore, there is a long way to go before these nanoparticles can be widely accepted and used. Moreover, we could focus on modifying nanoparticles, which augments their application and minimizes cell and organ toxicity. Finally, nanoparticles have mainly focused on PCa in terms of urinary tumors, and research in bladder cancer and kidney cancer is still insufficient. Further studies in these patients are also important and worthy of investigation. Thus, more efforts should be made to investigate the applications of nanoparticles, which we believe that we could lead to increasingly applicable and creative treatments for urinary tumors.

**TABLE 1:** Nanoparticle summary.

Nano particle	Key characteristic(s)	Advantages	Disadvantage(s)	Applications
MSNs	Mesoporous structures	High biocompatibility, adjustable particle size multifunctional surface, high loading capacity	Cytotoxicity, genotoxicity	Drug and gene delivery, MSN-assisted bioimaging, tissue regeneration, MSN-based carriers, MSN-based biosensors
AuNPs	Au	Large surface area, superior conductivity high biocompatibility, easy entry into the host	Cytotoxicity, organ toxicity neurotoxicity	Drug delivery, photothermal therapy, radiation therapy, immunotherapy, enzyme fixation, cell imaging
QDs	Quantum dot	High photochemical stability, fluorescence quantum yield and biocompatibility	Contain heavy metals, organ toxicity, environmental pollution, immunotoxicity	Biomolecule targeting, luminescence imaging, drug delivery
CNTs	Grapheme sheets	Small volume, high specific surface area, good cell penetration, can be combined with more biological macromolecules and drugs	Organ toxicity, genotoxicity	Biosensor, drug and gene delivery, vaccine delivery, tissue engineering, regenerative medicine, biomedical imaging, biosensors, biomolecular detection
Liposomes	Spherical structures	High biocompatibility, non-toxicnonimmunogenic, easy surface modification	Poor stability, low drug loading easy leakage	Drug delivery, increase drug stability, non-invasive imaging, drug targeting, gene therapy, tissue engineering
MNPs	Pure metals or their mixtures	High biocompatibility, magnetothermal effect, superparamagnetism	Cytotoxicity	Hyperthermia cancer treatment, controlled drug release, magnetic resonance imaging, biosensing, tumor imaging, radiotherapy
